# Characterization of the chromosomal inversion associated with the *Koa *mutation in the mouse revealed the cause of skeletal abnormalities

**DOI:** 10.1186/1471-2156-10-60

**Published:** 2009-09-22

**Authors:** Kentaro Katayama, Sayaka Miyamoto, Aki Furuno, Kouyou Akiyama, Sakino Takahashi, Hiroetsu Suzuki, Takehito Tsuji, Tetsuo Kunieda

**Affiliations:** 1 Graduate School of Natural Science and Technology, Okayama University, Okayama 700-8530, Japan; 2 Tokyo University of Agriculture and Technology, Tokyo 184-8588, Japan; 3 Laboratory of Veterinary Physiology, Nippon Veterinary and Life Science University, Tokyo 170-0071, Japan

## Abstract

**Background:**

Koala (*Koa*) is a dominant mutation in mice causing bushy muzzle and pinna, and is associated with a chromosomal inversion on the distal half of chromosome 15. To identify the gene responsible for the *Koa *phenotypes, we investigated phenotypes of *Koa *homozygous mice and determined the breakpoints of the inversion with a genetic method using recombination between two different chromosomal inversions.

**Results:**

Skeletal preparation of *Koa *homozygotes showed marked deformity of the ribs and a wider skull with extended zygomatic arches, in addition to a general reduction in the lengths of long bones. They also had open eyelids at birth caused by a defect in the extension of eyelid anlagen during the embryonic stages. The proximal and distal breakpoints of the *Koa *inversion were determined to be 0.8-Mb distal to the *Trsps1 *gene and to 0.1-Mb distal to the *Hoxc4 *gene, respectively, as previously reported. The phenotypes of mice with the recombinant inverted chromosomes revealed the localization of the gene responsible the *Koa *phenotype in the vicinity of the proximal recombinant breakpoint. Expression of the *Trsps1 *gene in this region was significantly reduced in the *Koa *homozygous and heterozygous embryos.

**Conclusion:**

While no gene was disrupted by the chromosomal inversion, an association between the *Koa *phenotype and the proximal recombinant breakpoint, phenotypic similarities with *Trps1*-deficient mice or human patients with *TRSP1 *mutations, and the reduced expression of the *Trsps1 *gene in *Koa *mice, indicated that the phenotypes of the *Koa *mice are caused by the altered expression of the *Trps1 *gene.

## Background

Many chromosomal rearrangements including translocation, insertion, deletion and inversion have been reported in mice, and they are often associated with developmental disorders [1]. In general, the developmental defects associated with chromosomal inversions are caused by disruption and/or alternation of expression of gene(s) located on or in the vicinity of breakpoints of the inversion [2,3]. Koala (*Koa*) is a dominant mutation of the mouse originating from X-ray irradiation to a C3H/HeH male mouse in the MRC Radiology Unit and it is associated with chromosomal inversion on the distal half of chromosome 15 [4,5]. *Koa *heterozygotes (*Koa*/+) have bushy muzzle and pinna, whereas *Koa *homozygotes (*Koa*/*Koa*) exhibit open eyelids at birth (EOB), dwarfism, and a flatter and broader head in addition to the phenotypic features of *Koa*/+ mice [4,5], but the exact phenotypes of *Koa*/*Koa *have not been described. Recently, Fantauzo *et al *[6] reported the localization of the breakpoints of the *Koa *inversion and reduced expression of *Trps1 *gene in a region close to the breakpoint, but exact assosiation between the *Koa *phenotype and the reduced expression of the *Trps1 *gene has not been confirmed.

*Eh *is another mutation of the mouse associated with a chromosomal inversion on the distal half of chromosome 15 overlapping with the inverted region of the *Koa *mutation [7,8]. We previously identified both proximal and distal breakpoints of the *Eh *inversion by a new genetic method that uses recombinant chromosomes generated by crossing-over between the inverted regions of the *Eh *and *Koa *mutations [9]. In the present study, we characterized the breakpoints of the chromosomal inversion of the *Koa *mutation and assigned the candidate region for the *Koa *phenotype by using the recombinant chromosomes to determine the gene responsible for the *Koa *phenotypes. We also investigated phenotypes of *Koa*/*Koa *mice including skeletal abnormalities, to further characterize the phenotypic features of the *Koa *mutation, which indicated phenotypic similarity between the *Koa *mouse and *Trps1*/*TRPS1 *deficient mice or humans. Our findings provide further evidence demonstrating that reduced expression of the *Trps1 *gene is responsible for the *Koa *phenotype.

## Results

### Phenotypes of the *Koa/Koa *mice

**Figure 1:**
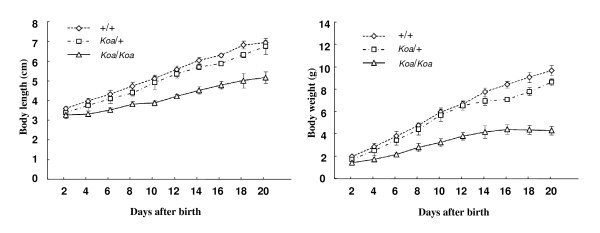
**Body length and weight of *Koa *mice**. Body length (left) and weight (right) of *Koa*/*Koa*, *Koa*/+, and +/+ mice were measured until 20 days after birth. Data are shown as the means ± SDs. The differences in body length were significant at postnatal days 4 to 20 between *Koa*/*Koa *and +/+ and days 14 to 18 between *Koa*/+ and +/+. The differences in body weight were significant at postnatal days 2 to 20 between *Koa*/*Koa *and +/+ and days 14 to 20 between *Koa*/+ and +/+. (P < 0.05).

Comparison of the body length and weight among different genotypes confirmed dwarfism in *Koa*/*Koa *mice. As shown in Figure 1, *Koa*/*Koa *mice were slightly smaller than +/+ and *Koa*/+ mice at post natal day 2 (P2) and the differences became more obvious in later stages. The average length and weight of the *Koa*/*Koa *mice at P20 were approximately 75% and 45% of those of +/+ mice, respectively. The *Koa/+ *mice were also slightly smaller than the +/+ mice.

**Figure 2:**
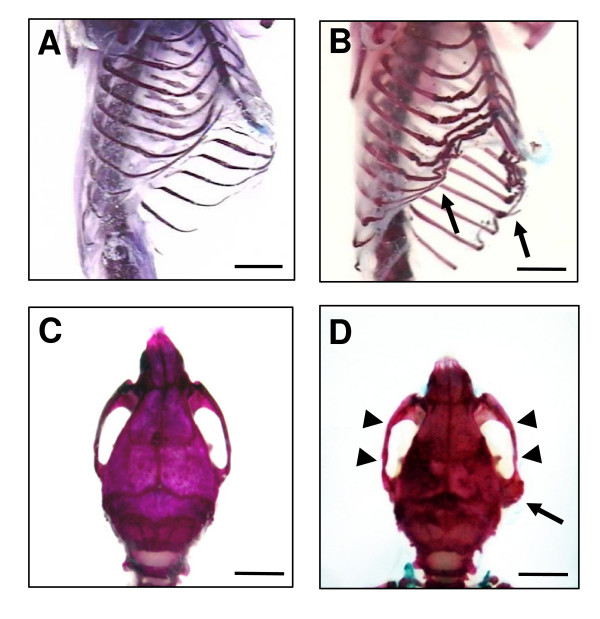
**Skeletal phenotypes of the *Koa*/*Koa *mice**. (A and B) Ventrolateral view of ribs of +/+ (A) and *Koa*/*Koa *(B) mice. Arrows indicate abnormal curvature of the ribs in *Koa*/*Koa *mice. (C and D) Skull of +/+ (C) and *Koa*/*Koa *(D) mice with dorsal views. Arrowheads and arrows indicate expanded zygomatic arches and deformed zygomatic processes of *Koa*/*Koa *mice, respectively.

The skeletal preparation of *Koa*/*Koa *mice showed that the shapes of the long bones of the limbs, digits, and vertebrae of *Koa*/*Koa *appeared to be normal, but the lengths of the long bones were significantly shorter than those of the +/+ mice (Table 1). Therefore, the dwarf phenotype observed in *Koa*/*Koa *mice may be due to a general reduction in the length of the long bones. However, the ribs and skull of *Koa*/*Koa *mice showed morphological abnormalities. As shown in Figure 2, marked deformities of costicartilage were observed in the ribs of *Koa*/*Koa *mice. The skulls of *Koa*/*Koa *mice were reduced in size along the anterior- posterior axis, but the left-right axes were almost the same as +/+ mice (Table 1). In particular, zygomatic arches were expanded and zygomatic processes were deformed in *Koa*/*Koa *mice (Figure 2). The expanded zygomatic arches are likely to be the cause of the flatter and broader shape of the head in *Koa*/*Koa *mice. Skeletal preparations of *Koa*/+ mice showed no apparent bone abnormalities including the ribs and skull (data not shown).

**Table 1:** Comparison of bone lengths between *Koa*/*Koa *and +/+ mice

			**Skull**		
			
**Genotype**	**tibia**	**femur**	**Length**	**Width**	**W/L**
*Koa/Koa *(n = 3)	15.9 ± 1.0*	12.7 ± 0.8**	20.9 ± 1.6*	13.6 ± 1.0	0.65 ± 0.02**
+/+ (n = 3)	18.3 ± 0.6	15.5 ± 0.4	24.4 ± 0.2	14.1 ± 0.05	0.58 ± 0.01

Values represent means ± SEM. *Statistically significant (*P *< 0.05). **Statistically significant (*P *< 0.005). W/L represents ratios of skull width and length.

**Figure 3:**
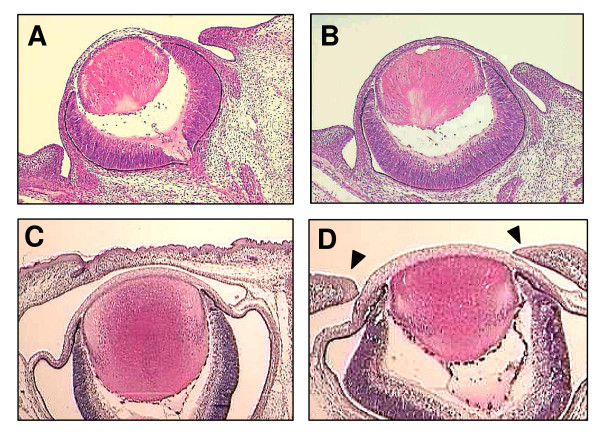
**Developing eyes of the *Koa*/*Koa *mice**. (A and B) Eyelids of E14.5 +/+ (A) and *Koa*/*Koa *(B) embryos. (C and D) Eyelids of E16.5 +/+ (C) and *Koa*/*Koa *(D) embryos. Arrowheads indicate eyelids that were not fused with each other.

Next, we examined the eyelids of newborn mice to confirm EOB of *Koa*/*Koa*. As shown in Table 2, 19 of 21 *Koa*/*Koa *mice showed EOB, while 2 of them were normal. In addition, 2 of 30 +/*Koa *mice showed EOB. Next, we examined the development of the eyelids in *Koa*/*Koa *embryos by histological examination. Anlagen of both the upper and lower eyelids of +/+ embryos formed at embryonic day (E) 13.5, grew at E14.5, and extended and fused with each other at E16.5. In *Koa*/*Koa *embryos, both eyelids' anlagen formed and grew at E 14.5, but did not extend and fuse with each other at E16.5 (Figure 3). Therefore, the EOB observed in *Koa*/*Koa *mice was caused by defects in the extension of the eyelid anlagen. In the present study, we also found a reduced number of vibrissae in the *Koa*/*Koa *mice (data not shown).

**Table 2:** Incidence of open eyelids at birth (EOB) phenotype in different genotypes

	**Bilateral EOB**	**Unilateral EOB**	**Normal**	**Total**
+/+	0	0	12	12
*Koa*/+	2	0	28	30
*Koa*/*Koa*	18	1	2	21

### Localization of the break points of the *Koa *inversion

**Figure 4:**
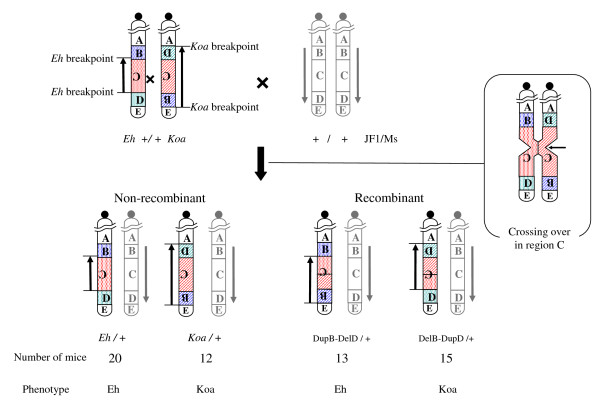
**Schematic diagram of recombinant chromosomes resulting from crossover between overlapping regions of *Koa *and *Eh *inversions**. The *Eh +*/+ *Koa *double heterozygote has both *Koa *type and *Eh *type chromosomes. If crossover occurred in the overlapping region of the two inversions (C region), recombinant chromosomes with duplication of the B region and deletion of the D region (DupB-DelD), or with deletion of the B region and duplication of the D region (DelB-DupD), were transmitted to the offspring. The phenotypes of mice with these chromosomes are indicated at the bottom.

**Figure 5:**
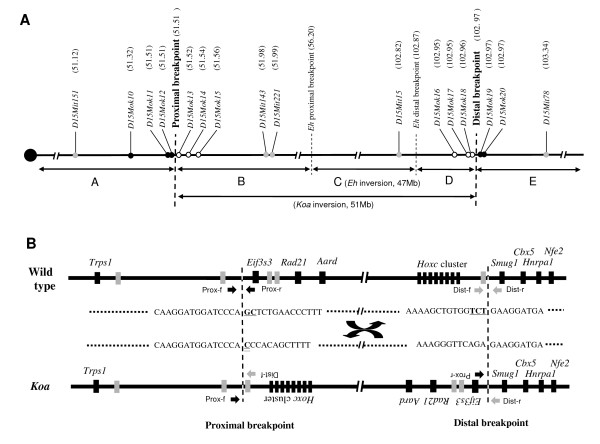
**Schematic diagram of the breakpoints of the *Koa *inversion**. **(A) **Map of mouse chromosome 15 showing positions of microsatellite markers surrounding the proximal and distal breakpoints. Numbers in parentheses indicate the position in the mouse genome sequence (Build 37.1). Markers showing hemizygosity and heterozygosity in mice with recombinant chromosomes are indicated by open and filled circles, respectively. Gray circles indicate markers used in the previous linkage analysis [8]. A, B, C, D and E represent the corresponding regions in Figure 4. **(B) **Genomic structure and nucleotide sequences of the breakpoints of the *Koa *inversion. Underlined and double underlined letters represent deleted and inserted nucleotides at the breakpoints, respectively. Filled and gray boxes represent functional genes and predicted transcriptional units with unknown function, respectively. Arrows indicate primers for cloning of the breakpoints.

We previously localized the proximal and distal breakpoints of the *Koa *inversion to regions between microsatellite markers *D15Mit151 *and *D15Mit143 *and between *D15Mit15 *and *D15Mit78 *on mouse chromosome 15, respectively [8]. In the present study, we employed a genetic method using a recombinant chromosome generated by crossing-over between the inverted regions of the *Eh *and *Koa *mutations, which has been described previously [9], to identify the exact breakpoints of the *Koa *inversion and the association between genes in the vicinities of the breakpoints and the *Koa *phenotype. We obtained 60 offspring from mating between *Eh *+/+ *Koa*, which possess both *Koa *and *Eh *chromosomes (Figure 4), and JF1/Ms. Due to the recombination between the *Koa *and *Eh *inverted regions, 15 of them had a deletion of the region flanked by the proximal breakpoints of the *Koa *and *Eh *inversions (B in Figure 4) and duplication of the region flanked by the distal breakpoints (D) (DelB-DupD in Figure 4), whereas 13 of them had a duplication of B and deletion of D (DupB-DelD) [8](Katayama et al., 2007, our unpublished data). It is notable that the DelB-DupD mice showed the *Koa *phenotype while the DupB-DelD mice showed the *Eh *phenotype. We genotyped 6 microsatellite markers (*D15Mok10-15*) located on a region close to the expected proximal breakpoint in these mice (Figure 5). The results indicated that *D15Mok13-15 *showed only the JF1 allele in the 15 DelB-DupD mice, while *D15Mok10-12 *showed heterozygosity of JF1/*Koa *or *Eh *alleles in all 60 mice. These findings indicate that *D15Mok13-15 *are localized in the deleted region (B), while *D15Mok10-12 *are localized on the outside of the *Koa *inversion. Therefore, the proximal breakpoint of the *Koa *inversion was localized within a 11-kb region between *D15Mok12 *and *D15Mok13 *(Figure 5). Next, we genotyped another 5 microsatellite markers (*D15Mok16-20*) located on a region close to the expected distal breakpoint. *D15Mok16-18 *showed only the JF1 allele in the 13 DupB-DelD mice, while *D15Mok19 *and *20 *showed heterozygosity of JF1/*Koa *or *Eh *alleles in all 60 mice. These findings indicated the localization of the distal breakpoint of the *Koa *inversion within a 5.2-kb region between *D15Mok18 *and *D15Mok19 *(Figure 5).

We then attempted a series of PCR amplifications in the 11-kb and 5.2-kb regions from +/+ and *Koa*/*Koa *genomic DNA. In the proximal region, 7 pairs of primers covering the entire 11-kb region were used for PCR amplification. Six of them amplified fragments with the expected size from both +/+ and *Koa*/*Koa *DNA. However, the one remaining fragment flanked by primers Prox-f and Prox-r (Figure 5 and Additional File 1) were not amplified from *Koa*/*Koa *DNA, while the fragment was amplified from +/+ DNA. Therefore, the proximal breakpoint was suggested to be localized in this fragment. Similarly, one of the 5 fragments in the distal region flanked by primers Dist-f and Dist-r (Figure 5 and Additional file 1) was not amplified from *Koa*/*Koa *DNA, suggesting the localization of the distal breakpoint in this fragment. To determine the exact sites of both breakpoints, we performed PCR amplification using primer pairs Prox-f and Dist-f, and Prox-r and Dist-r to amplify recombinant fragments containing the breakpoints (Figure 5) and their nucleotide sequences were determined. These pairs of primers amplified approximately 1-kb fragments from the *Koa*/*Koa *DNA, but not from +/+ DNA. The nucleotide sequences of these fragments revealed that the proximal and distal breakpoints were at 51,512,764 of the mouse chromosome 15 genome sequence (Build 37.1) in a region between the *Trps1 *and *Eif3s3 *genes, and at 102,965,601 in a region between the *Hoxc4 *and *Smug1 *genes, respectively (Figure 5). These results were concordant with those of Fantauzzo et al. [6]. Although there were 2- and 3-bp deletions and a 1-bp insertion at the breakpoints, no gene was disrupted by the *Koa *inversion (Figure 5).

As shown in Figure 4, the DelB-DupD mice showing the *Koa *phenotype had the proximal recombinant break point of the *Koa *inversion, but not the distal recombinant breakpoint, whereas the DupB-DelD mice showing the *Eh *phenotype had the distal recombinant breakpoint, but not the proximal recombinant breakpoint. This evidence indicated that the gene(s) responsible for the *Koa *phenotype is located in the vicinity of the proximal recombinant breakpoint and, therefore excluded the genes on the distal recombinant breakpoint from the candidate genes.

### Expression of genes in the vicinity of the breakpoints

**Figure 6:**
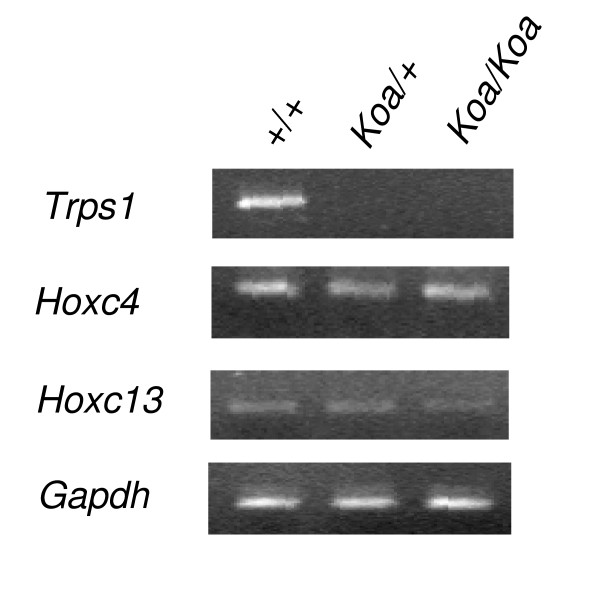
**Expression of candidate genes in mouse embryos**. Expression levels of *Trps1*, *Hoxc4*, and *Hoxc13 *genes were compared among E14.5 +/+, *Koa*/+, and *Koa*/*Koa *embryos. Expression of *Gapdh *was used as a standard.

To examine whether or not altered expression of the genes located near the proximal recombinant breakpoints is the cause of the *Koa *phenotypes, we compared expression levels of the *Hoxc4*, *Hoxc13*, and *Trps1 *genes, which are the most likely candidate genes for the phenotypes of the *Koa *mouse in these regions, among E14.5 +/+, *Koa*/+, and *Koa*/*Koa *embryos by semi-quantitative RT-PCR. While expression of *Hoxc4 *and *Hoxc13 *showed no apparent difference among these embryos, expression of *Trps1 *was markedly reduced in the *Koa*/+ and *Koa*/*Koa *embryos (Figure 6), indicating that the chromosomal inversion resulted in altered expression of the *Trps1 *gene.

## Discussion

In the present study, we confirmed the breakpoints of the *Koa *inversion by a genetic method using mice with the *Koa *and *Eh *recombinant chromosomes. Since no gene was disrupted by the *Koa *inversion, the phenotypes of the *Koa *mice were suggested to be caused by altered expression of gene(s) in the vicinity of the proximal and/or distal breakpoints. Next, we excluded the genes on the distal recombinant breakpoint from the candidate genes by association between the *Koa *phenotypes and the proximal recombinant breakpoints in DelB-DupD and DupB-DelD mice. As shown in Figure 4, there are at least 10 functional genes (*Trps1 *and *Hoxc4*, *5*, *6*, *8*, *9*, *10*, *11*, *12*, *13*) and several predicted transcriptional units in the vicinities of the proximal breakpoint. However, the reported functions of the known genes or phenotypes of the mice with disruption of these genes indicated that no genes except for the *Hoxc4*,*Hoxc13 *and *Trps1 *genes were likely to be involved in the phenotypes of the *Koa *mouse. The targeted disruptions of the *Hoxc4*,*Hoxc13 *and *Trps1 *genes were reported to cause defects in the thoracic vertebrae, alopecia, and abnormalities in the vibrissae, skull and thoracic vertebrae, respectively [10-12]. Therefore, we examined the expressions of these genes and found that the expression of the *Trps1 *gene was significantly reduced in the *Koa*/*Koa *and *Koa*/+ embryos while no significant difference was observed in the *Hoxc4 *and *Hoxc13 *genes. These findings strongly suggested that the reduced expression of the *Trsp1 *gene caused by chromosomal inversion is responsible for the phenotypes of the *Koa*/*Koa *mice.

The *Trps1 *gene encodes one of the GATA transcriptional factors defined by the presence of zinc finger motifs with a consensus sequence and has essential functions in vertebrate development [13-16]. *Trps1 *is widely expressed during embryogenesis, including expression within developing ribs, limbs, digits, vertebrae, snout, hair follicles and skin surrounding the eyes [17,18], and regulates chondrogenesis [19-21]. Mutations of *TRPS1 *in humans cause autosomal-dominant hereditary diseases, tricho-rhino-phalangeal syndromes (TRPS) type I and III, and patients with TRPS exhibit craniofacial malformation, bone abnormalities and sparse scalp hair [22-24]. In addition, targeted disruption of the *Trps1 *gene in the mouse causes craniofacial abnormalities, kyphoscoliosis and reduced trabecular bone in heterozygous mice, and a lack of vibrissae, scoliosis and abnormalities in the thoracic spine and ribs in homozygous mice [12]. In the present study, we found that *Koa*/*Koa *showed skull and rib deformities and a reduced number of vibrissae hair follicles, which resembled the phenotypes of the *Trps1*-deficient mice. These phenotypic similarities also suggested the involvement of *Trps1 *in the *Koa *phenotypes. In addition, the impaired extension of the eyelids of *Koa*/*Koa *mice can be explained by the function of the *Trsp1 *gene, since *Trps1 *is expressed in the skin surrounding the eyes during embryonic development [17].

Although localization of the breakpoints of the *Koa *inversion and reduced expression of *Trsp1 *have been reported by Fantauzzo et al. [6], they could not rule out the possibility that the other genes in the vicinities of the breakpoints are responsible for the *Koa *phenotype. However, our findings including an association between the *Koa *phenotype and the proximal recombinant breakpoint, the normal expression of the *Hoxc4 *and *Hoxc13 *genes in *Koa *mice, and phenotypic similarities with *Trps1*-deficient mice or human patients with *TRSP1 *mutations, provide novel evidence indicating that the *Trps1 *gene, but not the other genes in the vicinities of the breakpoints, is responsible for the *Koa *phenotype.

In addition to the skeletal anomalies, the *Koa*/+ and *Koa*/*Koa *mice showed extra hair on the pinna and a bushy muzzle, which were not reported in the *Trps1*-deficient mice. Since the proximal breakpoint of the *Koa *inversion is located approximately 0.8-Mbp apart from *Trps1*, these differences in the phenotypes between *Koa *and *Trps1*-deficient mice could be reflected by the different types of mutations. Chromosomal rearrangements often affect the expression pattern of the genes located near the breakpoint by a position effect. The position effects of *cis*-regulatory elements on transcription of genes have been well documented in mutant mice involving the *Shh *gene [25-27]. Mice having mutations or chromosomal rearrangements involving the *cis*-regulatory elements of the *Shh *gene share several phenotypic features similar to those of *Shh*-deficient mice, but some of them also exhibit phenotypes not observed in *Shh*-deficient mice. These differences are thought to be caused by ectopic expression of *Shh *during embryogenesis [3,27]. Therefore, the differences in the phenotypes between the *Koa *mice and *Trps1*-deficient mice may be due to the ectopic expression of *Trps1 *during embryogenesis caused by chromosomal inversion.

It is notable that almost no expression of the *Trps1 *gene was observed not only in the *Koa*/*Koa *homozygous mice, but also in the *Koa*/+ heterozygous mice (Figure 1), although they have one wild-type chromosome. This unexpected level of *Trps1 *expression in *Koa*/+ mice is the same as that reported in Fantauzzo *et al*. [6] and is concordant with the dominant mode of inheritance of the *Koa *mutation. One possible mechanism for the dominant suppression of the *Trps1 *expression in *Koa *mice is that transcription of antisense RNA of the *Trps1 *gene is enhanced by the chromosomal inversion resulting in suppression of the sense *Trps1 *transcription from both mutant and wild-type chromosomes. Tufarelli *et al*. reported that expression of antisense RNA caused by a deletion in a region close to the α-globin gene resulted in methylation of a nearby CpG island and silencing of the α-globin gene expression [28]. A similar mechanism may be involved in the dominant suppression of *Trps1 *expression in the *Koa *mice.

## Conclusion

In the present study, we found skeletal abnormalities in *Koa*/*Koa *mice and determined the breakpoints of the *Koa *chromosomal inversion. While no gene was disrupted by the chromosomal inversion, expression of the *Trsps1 *gene, which is localized close to the proximal breakpoint, was significantly reduced in *Koa *mice. The present findings including the association between the *Koa *phenotype and the proximal recombinant breakpoint, phenotypic similarities with *Trps1*-deficient mice or human patients with *TRSP1 *mutations, and the reduced expression of the *Trsps1 *gene and normal expression of *Hoxc4 *and *Hoxc13 *genes, indicated that the phenotypes of the *Koa *mice, at least to some extent, are caused by altered expression of the *Trps1*gene.

## Methods

### Animals

*Koa *and *Eh *mutant mice were obtained from the Medical Research Council (Harwell, Oxfordshire, UK) and the Jackson Laboratory (Bar Harbor, Maine, USA), respectively. JF1/Ms mice were obtained from the National Institute of Genetics (Mishima, Japan). The genotypes of the mice were determined by PCR using primers flanking the breakpoints of the inversion (Additional File 1). Body length from the nose to the anus and body weight of 5 +/+, 5 +/*Koa*, and 3 *Koa*/*Koa *mice were measured every 2 days until P20. All data are expressed as the means ± SD, and the statistical significance of differences was determined using the Student's t-test.

### Skeletal and histological preparations

*Koa*/*Koa*, *Koa*/+, and +/+ adult mice were used for skeletal preparation. The skin and internal organs of the mice were removed. The skeletons were fixed in 95% ethanol for 1 day and then stained with 0.15% alcian blue in 80% ethanol and 20% acetic acid for 1 day. Fixed skeletons were dehydrated in 100% ethanol and immersed in 2% KOH for 1-7 days. The skeletons were then stained with 0.015% alizarin red in 1% KOH for 1 day, cleared in a series of graded glycerin, and stored in glycerin and ethanol (1:1). Mouse embryos were fixed in 4% paraformaldehyde for 16-24 hrs at room temperature. After dehydration, the embryos were embedded in paraffin and sectioned at 4 μm thickness. Hematoxylin and eosin-stained sections were observed under a light microscope.

### Generation of recombinant chromosomes and localization of the breakpoints

*Koa*/+ mice were mated with *Eh*/+ mice to obtain *Koa *+/+ *Eh *mice, which have both the *Koa *and *Eh *inversions. The *Koa *+/+ *Eh *mice were then mated with mice of the JF1/Ms strain, and 60 offspring were obtained. Genomic DNAs were prepared from mouse livers by phenol/chloroform extraction. To determine the deleted or duplicated region of the recombinant chromosomes, the genotypes of 11 new microsatellite markers obtained from the genomic sequence of this region were determined. The genotypes were obtained by PCR amplification in a reaction mixture containing 20 ng of genomic DNA, 0.2 mM dNTP, 0.2 μM of each primer and 0.25 U of Taq DNA polymerase (Amersham Bioscience, Piscataway, NJ, USA) with 35 cycles at 94°C for 30 sec, 50-60°C for 30 sec and 72°C for 30 sec. The PCR products were electrophoresed on 3.0% agarose gel and stained with ethidium bromide. Primer information for these microsatellite markers is shown in Additional file 1. The exact breakpoints of the inversion were determined by a series of PCR amplifications of 1- to 2-kb fragments in the critical region. The PCR products were cloned into pGEM-T Easy vector (Promega, Madison, WI, USA) and sequenced by an ABI310 automated sequencer.

### Expression analysis of genes in the vicinities of the breakpoints

E15.5 embryos of each genotype were obtained from mating between *Koa*/+ mice. Total RNA was extracted from the embryos and reverse transcribed using Superscript III reverse transcriptase (Invitrogen, Carlsbad, CA, USA) with random hexamers for 60 min at 42°C. The expression of candidate genes in the vicinities of the breakpoints was examined by semi-quantitative RT-PCR under the following conditions: 30 - 40 cycles consisting of denaturation at 94°C for 30 sec, annealing at 50-55°C for 30 sec, and extension at 72°C for 30 sec. Primer information for the RT-PCR is shown in Additional file 1.

## Authors' contributions

KK carried out the molecular genetic studies and drafted the manuscript. SM participated in molecular genetic studies and carried out morphological studies. AF participated in molecular genetic and morphological studies. KA and TT participated in mouse breeding and sequence alignment. ST and HS helped to draft the manuscript. TK conceived the study, carried out its design and coordination, and helped to draft the manuscript. All authors read and approved the final manuscript.

## Supplementary Material

Additional file 1 **Supplemental table S1**. Nucleotide sequences of primers for PCR. Click here for file
