# Supplementary material for: Characterization of the chromosomal inversion associated with the Koa mutation in the mouse revealed the cause of skeletal abnormalities
Source: BMC Genet. 2009 Sep 22;10:60. doi: 10.1186/1471-2156-10-60 (PMC2758895; doi:10.1186/1471-2156-10-60)
Supplement: Additional file 1 — Supplemental table S1. Nucleotide sequences of primers for PCR. [file 1471-2156-10-60-S1.DOC]

**Supplemental Table 1. Nucleotide sequences of primers for PCR**

^1^Primers for microsatellite markers

| Name of  primers | Primer Sequences | |
| --- | --- | --- |
|  | Forward (f) | Reverse (r) |
| *D15Mok10*^1^  *D15Mok11*^1^  *D15Mok12*^1^  *D15Mok13*^1^  *D15Mok14*^1^  *D15Mok15*^1^  *D15Mok16*^1^  *D15Mok17*^1^  *D15Mok18*^1^  *D15Mok19*^1^  *D15Mok20*^1^  Prox-f, -r^2^  Dist-f, -r^2^  Trps1^3^  Hoxc4^3^  Hoxc13^3^  Gapdh^3^ | 5'-TATCTGAGAATCTCCTGCTCAGAC-3'  5'-ACAACTTAAGGCCCATGAGAGGAT-3'  5'-GGTAAAACAGAGTAAGCCTATGGC-3'  5'-CAGGATCAACATGGCAGAATCCC-3'  5'-CTTTTCTGTGAACCACAGTGGATG-3'  5'-CTGGCTACAGCTCAAGAGCACT-3'  5'-TGATCATATGTCTGGATGGGCATG-3'  5'-GGATGCTGTGAAAGAGTCTGTCTA-3'  5'-CTGCAGAAGCAGACTGGAGTTAG-3'  5'-CTGACAGGGAACTTTCTGCCTCT-3'  5'-CTCACAGTGAGACTGAGAATCTTAA-3'  5'-GGTAAAACAGAGTAAGCCTATGGC-3'  5'-TTTGCACGCCGAGAAACACACTG-3'  5'-CAGTTCCCGAGAGCATAGTCAAG-3'  5'-CCCGGCCCGGCGCTGCGCCCAGCAC-3'  5'-CCCGTCCGTTTGCCCGCCCT-3'  5'-CTTTGGCATTGTGGAAGGG-3' | 5'-TGCGCACACACATGTGGGG -3'  5'-CTTCACTAAAATGAATGTCATCATGGT-3'  5'-TTTTGAAGACAAACACCAGTTTCAGA -3'  5'-GGGAGGAGGGTATAGGGGATTTT -3'  5'-TGGCAGATAGCATCTCAAAGCAAC-3'  5'-GATAAATTCTGATACGTCTCTCCAT-3'  5'-AGCCAGGACTACACAGAGAATCTC-3'  5'-ATTGCCAGAATACCCATTCCTTGG 3'  5'- AGCCTGCTTCCAACCAAACTACC-3'  5'-CTCTGCTGGAATCTAAGTCCACAT-3'  5'-GAGCCAGGTGAGAAGATACCTGAA-3'  5'-ATCAGCAGTGGGGAGGTTATGGT-3'  5'-GCAGAGCACTTCCTGAGCTAATG-3'  5'-CTGCTCAGCCTGAAGTGCCTCTG-3'  5'-CCAGCAGGTACCATGCTAAGACAAC-3'  5'-GGCAAGAACTGAGTAATTTGG-3'  5'-CCTCTCTTGCTGCAGTGTC-3' |

^2^Primers used for cloning of the breakpoints and *Koa* genotyping

^3^Primers used for semi-quantitative RT-PCR
